# Eco-Environmental Effect Evaluation of *Tamarix chinesis* Forest on Coastal Saline-Alkali Land Based on RSEI Model

**DOI:** 10.3390/s22135052

**Published:** 2022-07-05

**Authors:** Jin Wang, Guangxue Li, Feiyong Chen

**Affiliations:** 1 Research Institute of Resources and Environment Innovation, Shandong Jianzhu University, Jinan 250101, China; wangjin21@sdjzu.edu.cn; 2 Key Lab of Submarine Geosciences and Prospecting Techniques, Ministry of Education, Ocean University of China, Qingdao 266100, China; estuary@ouc.edu.cn

**Keywords:** remote sensing, tamarix forest, eco-environment effect, comprehensive evaluation, spatial analysis, Changyi, Shandong, China

## Abstract

Taking representative *Tamarix chinensis* forest in the national-level special protection zone for ocean ecology of Changyi city in Shandong province of China as the objective, this research studied how to use remote sensing technology to evaluate natural eco-environment and analyze spatiotemporal variation. In the process of constructing the index system of ecological environment effect evaluation based on RSEI (Remote Sensing Ecological Index) model, AOD (Aerosol Optical Depth), Salinity, Greenness, Wetness, Heat and Dryness, which can represent the ecological environment of the reserve, were selected as the corresponding indexes. In order to accurately obtain the value of the RSEI of the study area and to retain the information of the original indexes to the greatest extent, the SPCA (spatial principal components analysis) method was applied in this research. Finally, the RSEI was applied to evaluate the ecological and environmental effects and to analyze the spatial characteristics and spatiotemporal evolution of the study area. The results not only provide scientific evidence and technical guidance for the protection, transformation and management of the *Tamarix chinensis* forest in the protection zone but also push the development of the universal model of the ecological environment quality with a remote sensing evaluation index system at a regional scale.

## 1. Introduction

With the continuous development of society, there is gradually more attention on natural eco-environment quality and higher requirements on the timeliness, continuance, standard, scale and technical specification of the eco-environment quality monitoring and evaluation. Without doubt, this also offers opportunities for the application of remote sensing technology in the eco-environment quality monitoring and evaluation [1]. Until now, the objectives of the environmental quality monitoring and evaluation most belong to large to medium scale, but less on small scale, which regards the partial area as a unit. Simultaneously, the index of existing eco-environment evaluation is always goal-oriented and has regional characteristics, making it hard to promote this evaluation. In addition, the traditional monitoring and evaluation system, in most cases, takes advantage of one value to represent the ecological situation of the entire research region, so it lacks continuance and visibility, finally making it hard to deeply understand and analyze the spatial difference and dynamic change of regional ecological quality [2]. Remote sensing technology has the characteristics of rapid, dynamic, continuous, quantitative, repeatable and objective monitoring. Besides, it is of the ability to express the results of eco-environment monitoring and evaluation in the form of visualizing, modeling and forecasting of spatiotemporal variation on the basis of GIS or RS processing platforms [3]. In recent years, remote sensing technology has been applied to eco-environmental factor extraction, ecological landscape variation analysis, ecological risk evaluation, eco-environmental evolvement and driving factor analysis [4].

*Tamarix chinensis* belongs to the shrub or dungarunga of tamarix (*Tamaricaceae*) and has some biological characteristics of strong salt tolerance, drought resistance and flood resistance. Now, it is widely distributed in inland saline land, northern coastal wetland and beach land of China. *Tamarix chinensis* plays a significant role in the construction of ecology and forestry. According to a previous study, it is indispensable in eco-environment maintenance, water conservation, remediation of saline-alkali soil, and the greening of saline-alkali land [5]. In 2005, through a forestry investigation into the coastal area of Laizhou Bay, the forestry bureau of Changyi city in Shandong province of China found the natural *Tamarix chinensis* forest, which had different distribution densities, the biggest continuous area, typical structure and complete preservation, in the northern coastal area of Changyi city. In October 2007, the State Oceanic Administration of China approved the establishment of Shandong Changyi’s national marine ecological special reserve (hereinafter referred to as a ‘protection zone’ in the following). Now, the protection zone is the only national-level special one for ocean ecology that takes *Tamarix chinensis* as the main protection objective, so it is representative to select its *Tamarix chinensis* forest for study.

By referring to relevant literature, it was found that there was little research on how to apply remote sensing technology to the eco-environment monitoring and analysis of the *Tamarix chinensis* forest or its adjacent area of the protection zone. Hence, this research took the *Tamarix chinensis* forest in the protection zone as the study area, to study the way of how to use remote sensing technology for the evaluation and the spatiotemporal variation analysis of the natural eco-environment. The obtained results not only provide scientific evidence and technical guidance to the protection, transformation and management of the *Tamarix chinensis* forest in the protection zone but also push the development of the universal model of the eco-environment quality with a remote sensing evaluation index system at a regional scale.

## 2. Study Area and Data

**Figure 1:**
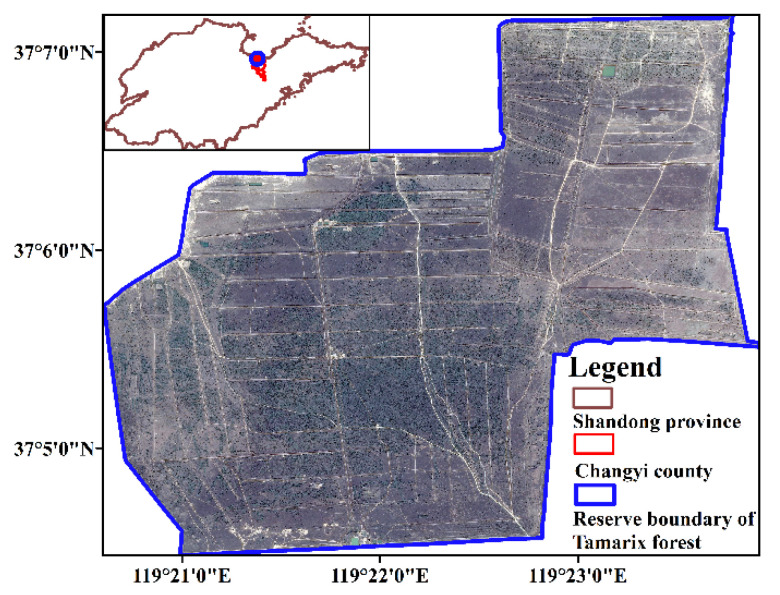
Location of study area.

The *Tamarix chinensis* forest in the study area starts from a damp-proof dam of National Defence University’s salt field in the east, extends to the Di River along the embankment in the west, reaches the branch of the Di River along the embankment in the south, and shares a border with the shallow seaside area in the north. The research area, approximately 1548 hm^2^, is 5 km wide from east to west and 7 km long from north to south [6]. The geographical location of the study area is shown in Figure 1.

For reflecting more comprehensive and high-accuracy of the spatial distribution characteristics and the current condition of the eco-environment of the protection zone, this research chose the Senitnel-2A-MSI (L1C) image, which was taken in September 2019 and had high spatial resolution and an abundant spectrum. At the same time, it also selected the HJ1-CCD remote sensing image data to reflect the spatial distribution of AOD in the protection zone. Additionally, four historical remote sensing images of the Landsat series, which were taken from September to October with small cloud coverage coefficients, demonstrate the eco-environment situation during the crucial periods and the related dynamic change trend of the last 20 years in the protection zone. In Table 1, it lists time points and selects reasons for the selected images. 

## 3. The Construction of Remote Sensing Index System of Eco-Environment Evaluation

In 2006, the State Environmental Protection Administration of China (SEPA) issued Technical Specification for Eco-environment Condition (HJ/T 192-2006) (referred to as ‘specification’ in the following) in the form of an industry-standard and proposed ecological index (EI) based on remote sensing technology. Later, after the amendment in 2015, the specification added special eco-environment evaluation factors and calculation methods towards ecological function area, city/city cluster and natural protection zone, aiming to provide a type of annual comprehensive eco-environment evaluation standard for the region at no less than the county level. The specification mainly contains the following five eco-environment evaluation factors: biological abundance, vegetation fractional coverage (VFC), water network density, environmental quality and land degradation. The final regional eco-environment index was built through the weighted summation of these five factors. 

From specification, EI is appropriate for the evaluation of the region at no less than the county level but restricted to smaller regions, such as towns, small watersheds and natural protection zones. Besides, affected by the data collection of environmental quality factors, evaluation is carried out only once per year, leading to obviously limited timeliness. To avoid this limitation and carry out an eco-environmental evaluation of the protection zone with remote sensing technology as the core, this research proposed six factors to construct the remote sensing ecological evaluation index system. These six factors were the following: Aerosol Optical Depth (AOD), Salinity, Greenness, Wetness, Heat and Dryness. These six factors had near timeliness and the ability to represent the eco-environment of the protection zone. Among them, the last four factors are not only important factors of natural ecological quality but also are closely related to human activities. Therefore, these four factors are also important factors for humans to evaluate the quality of the ecological environment [7]. In addition, the protection zone belongs to the coastal *Tamarix chinensis* wetland, and its land cover is of forest type. So, the factors of salinity and AOD were selected as the evaluation index systems [8]. In terms of data acquirement, it is possible to directly or indirectly extract these six factors from remote sensing images with the fast development and gradual maturity of remote sensing technology. Therefore, the constituted RSEI in this research was scientific and reasonable, and RSEI can be regarded as the function of these six factors, namely, RSEI = F (AOD, Salinity, Greenness, Wetness, Heat, Dryness). 

### 3.1. AOD

Aerosol, a suspension of liquid or solid particles dispersed in air or gas, circulates in many atmospheric chemical cycles and is an important component of the atmospheric environment. In addition, aerosols have obvious atmospheric environmental effects, with an example of PM_2.5_ aerosol, which has an extinction effect on light. Because of eco-environment deterioration, there are more and more haze events in the local area caused by PM_2.5_ pollution [9]. PM10 aerosol is regarded as a carrier and catalyst for many pollutants, so it is seriously harmful to human health [10]. As one of the most fundamental optical characteristics of aerosol, AOD becomes a significant parameter for studying atmospheric turbidity and is capable of reflecting aerosol distribution change to some extent. In practice, AOD distribution is affected by the geographical environment, population density and industrial distribution, so it can display atmospheric turbidity and pollution [11]. When AOD increases, the optical thickness of aerosol grows and the atmospheric transmissivity decreases; otherwise, optical thickness decreases and transmissivity grows. In addition, a previous study has demonstrated that the AOD of forest and grassland with high vegetation coverage is lower than that of cities and towns where human activities are frequent, which means that forest is able to reduce AOD [12]. 

It is difficult for traditional observation to reflect the spatial distribution and change of aerosol. By contrast, aerosol monitoring based on remote sensing technology is featured by low cost, a wide range and the capability of reflecting spatial distribution and change, so it has become an important method in aerosol monitoring [13]. Thus, this research inversed AOD spatial distribution by remote sensing technology and analyzed the atmospheric environmental effects of the *Tamarix chinensis* forest in the protection zone.

#### 3.1.1. The Theory of Dense Dark Vegetation Method

With the help of optical remote sensing satellites, there are two common algorithms used to inverse the optical thickness of terrestrial aerosol. The two methods are the dense dark vegetation (DDV) method and the deep blue algorithm, respectively. The DDV method takes advantage of the low surface reflectivity of dense vegetation in the red band and blue band to constitute the linear relationship of dark pixels in these two bands. Then, the surface reflection contribution is removed from the satellite observation signal for aerosol information extraction [14]. In the consideration of the actual situation in the protection zone, this research selected the DDV method to inverse AOD. 

Through a large number of experimental data, Kaufman concluded that, in areas of dense vegetation, there was a strong correlation between the surface reflectivity of middle-infrared at 2.1 μm and the surface reflectivity (ρ) of the blue band and red band at 0.47 μm and 0.66 μm, respectively [15]. As shown in the following:

(1) $$\rho_{0.66}=\frac{1}{2}\;\times \;\rho_{2.1}$$

(2) $$\rho_{0.47}=\frac{1}{4}\;\times \;\rho_{2.1}$$

Moreover, because the middle-infrared band at 2.1 μm is seldom affected by atmospheric aerosol, apparent reflectivity ($\rho_{2.1}^{*}$) is approximately equal to surface reflectivity ($\rho_{2.1})$.

(3) $$\rho_{0.66}=\frac{1}{2}\;\times \;\rho_{2.1}=\frac{1}{2}\;\times \;\rho_{2.1}^{*}$$

On the assumption that the surface of the target observed by satellite is the uniform Lambert surface and gas absorption is ignored, the apparent reflectivity observed by satellite is as follows:

(4) $$\rho^{*}\left(\theta_{s},\theta_{v},Ø\right)=\rho_{a}\left(\theta_{s},\theta_{v},Ø\right)+\frac{\rho }{1-S*\rho }\;\times \;T$$

In Equation (4), $\theta_{s},\theta_{v}\;\mathrm{and}\;Ø$ refer to Solor Zenith, Satellite Zenith and Azimuthal Angel Difference, respectively.

**Figure 2:**
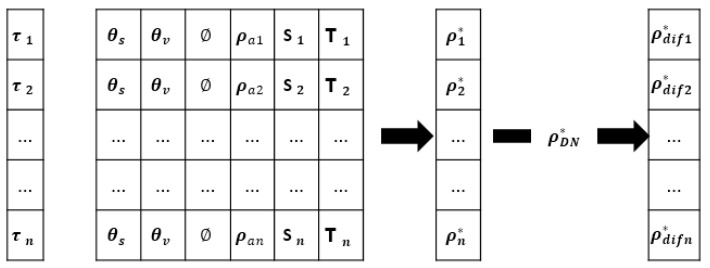
The principle of 6S model.

Hence, $\rho^{*}$ is the function of not only AOD but also underlying surface reflectivity. Through the Second Simulation of the Satellite Signal in the Solar Spectrum (6S) atmospheric radiation transmission model, it is possible to calculate the corresponding $\rho_{\alpha }$ (reflectivity of atmospheric molecules and aerosols), *S* (atmospheric spherical albedo) and T (total atmospheric transmissivity) under different AOD. Therefore, after confirming the surface reflectivity of the visible light channel and reasonably assuming the aerosol model, it is possible to obtain the 6S model and then calculate the apparent reflectivity of this channel. Under the comparison of the 6S models’ apparent reflectivity with remote sensing images, it is able to inversely deduce the actual AOD [16]. The theory of using the 6S model to inverse AOD can be found in Figure 2. 

In short, the inversion process of aerosol can be divided into the following three parts: the data acquisition and preprocessing of the satellite image, the construction of the lookup table used for aerosol inversion, and the inversion and analysis of AOD distribution in terms of the 6S model. 

#### 3.1.2. Data Acquisition and Data Preprocessing of HJ1-CCD

This research took HJ1-CCD data as source data in the AOD inversion. During preprocessing, the radiometric calibration of the image was performed according to the calibration coefficient from the experiment, then the digital number (DN) of the image was transformed into radiant brightness. Next, the solar elevation angle and imaging time were read from the metadata information of the image data and finally, apparent reflectivity was calculated.

#### 3.1.3. Lookup Table Construction

In the actual inversion process, the lookup table solves atmospheric radiation by means of the radiation transmission model. In general, the first is to confirm atmospheric parameters, secondly, it is to select different AODs according to solar incident angles and satellite observation angles.

It should be added that, in Table 2, $\theta_{s},\theta_{v}\;\mathrm{and}\;Ø$ refers to Solar Zenith, Satellite Zenith and Azimuthal Angel Difference, respectively. The specific values of each parameter in this research are as follows:

Solar Zenith: 0°, 5°, 10°, 15°, 20°, 25°, 30°, 35°, 40°, 45°, 50°, 55°, 60°, 65° and 70°.

Satellite Zenith: 0°, 5°, 10°, 15°, 20°, 25°, 30°, 35° and 40°.

Relative Azimuth: 0°, 12°, 24°, 36°, 48°, 60°, 72°, 84°, 96°, 108°, 120°, 132°, 144°, 156°, 168° and 180°.

Lastly, the 6S model is used to calculate the reflectivity received by satellites under various conditions and to make the lookup table used for AOD inversion. Based on the atmospheric model, aerosol type, the geometric parameters of satellite observation and AOD, it is able to construct a lookup table corresponding to different sensors, which is composed of *S*, $\rho_{\alpha }$, and T. The lookup table for this research is shown in Table 2.

#### 3.1.4. The Calculation and Output of AOD

In the application of the DDV method, it is able to confirm dark pixels through setting the NDVI threshold. This research obtained NDVI by calculating the atmospheric correction image and setting the pixel under the condition of NDVI > 0.3 as a dark one according to the actual condition of the protection zone [17]. In terms of an atmospheric model, aerosol type, solar zenith angle and other parameters, the constructed lookup table was interpolated. Additionally, we could obtain the three atmospheric parameters (S, $\rho_{\alpha }$ and T) of the dark pixel in the red band and blue band under different AOD values. Next, the research put the three parameters and the apparent reflectivity of the top atmosphere into formula (4) to obtain the surface reflectivity at the red band and blue band under different AOD values. Because there was no band at 2.1 μm in the HJ1-CCD sensor, it was necessary to respectively compare the ratio between the surface reflectivity of the red band and the surface reflectivity of the blue band with the set ratio (k) for the corresponding sensors. The minimum difference (absolute value) corresponded to the inversed AOD. When the HJ1-CCD sensor was selected, k was set to 1.60 [18]. After computing the AOD of dark pixels, the research used interpolation to calculate the AOD of the non-dark pixels through the distance weighted average method, then processed the computed results by smooth effect and finally output the AOD result of the protection zone after adding the projection information.

#### 3.1.5. AOD Result and Analysis

**Figure 3:**
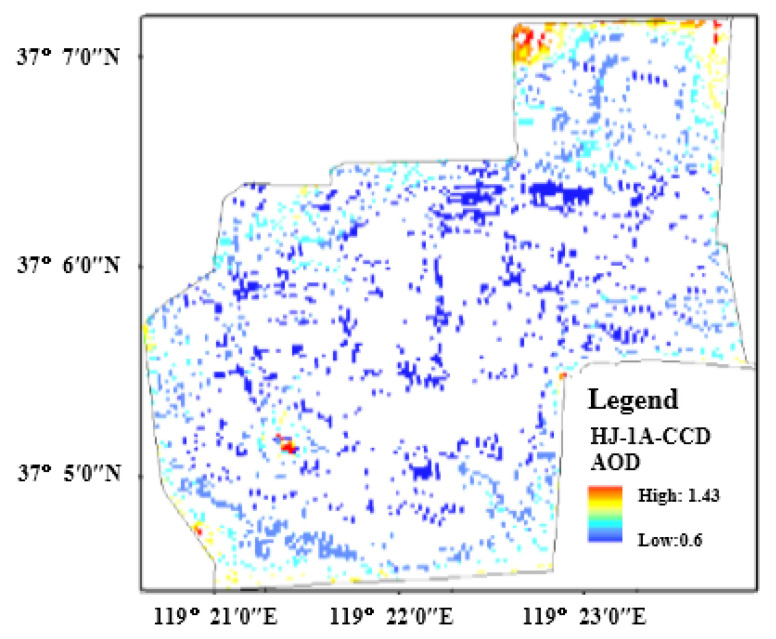
The inversion results of AOD in the study area based on HJ1A-CCD data.

Through the analysis of Figure 3, it can be seen that the inversed AOD has a highly precise spatial resolution of 30 m. In the protection zone, the AOD value of the *Tamarix chinensis* forest ranges from 0.50 to 1.43, and the AOD in the marginal area is distinctly higher than that in the middle area. In accordance with inversion results, AOD was relatively high in September 2019. According to on-site research, we analyzed that AOD value was related to urbanization, industrial construction development, population density and the poor eco-environment in the protection zone and its surroundings [19]. 

### 3.2. Salinity 

A field investigation found that the soil in the protection zone was saline soil. Practically, it plays an important role in the management of saline soil, the prevention of soil deterioration and ecologically sustainable development to obtain real-time and reliable information about the property, range, area, geographical distribution and salinization degree of saline soil. Now, due to the macro, comprehensive, dynamic, fast and other features of remote sensing technology, it has become an emerging method to monitor soil salinization [20]. 

On the basis of the remote sensing data from Sentinel-2A on 9 September 2019 and the field sampling data, which were synchronous with the remote sensing time of the satellite, this research built the inversion model of the correlation between the spectrum information and soil salinity. In this research, the soluble salt content of soils was used to denote soil salinity.

#### 3.2.1. Characteristic Bands Selection

To select the characteristic bands which could quantitatively inverse the soil salt content in the protection zone, after the preprocessing of clipping, atmospheric radiation correction and geometric accurate calibration, the measured data, including the reflectivity of each band and the test data of the soluble salt content of soils of 10 training sample points, were processed with correlation and discreteness analyses. 

From Table 3, the soluble salt content of soils is positively correlated with the reflectivity of the Sentinel-2A remote sensing image in the blue, green and red bands of visible light and SWIR band. However, the correlation is negative between the reflectivity of vegetation in the red edge band, the NIR band and the soluble salt content of soils. Especially in the bands of 6, 7, 8 and 8A, the reflectivity has a better correlation with the soluble salt content of soils than that in the other bands. In addition, the mean square deviation is relatively small in the blue, green and red bands of visible light and the shortest B5 band of vegetation at the red edge.

In order to further study the sensitivity between the reflectivity of different bands and the soluble salt content of soils, the diagnosis index (Di) was introduced into this research [22]. For the differences in sensitivity between the reflectivity from the Sentinel-2A satellite multispectrum sensor and the soluble salt content of soils, the calculation formula of Di is as follows:

D_i_ = 100 × σ_i_ × R_i_ (5)

where, σ_i_ is the mean square deviation of the reflectivity at the i band, and R_i_ refers to the correlation coefficient between the reflectivity at the i band and the soluble salt content of soils. When the diagnosis index is relatively large, the corresponding band represents the diagnosis and characteristic band of soil salt content. Detailed results are shown in Table 4. In Table 4, the diagnosis index is larger at 2, 6, 7, 8 and 8A bands than the others in the Sentinel-2A multispectrum remote sensing image, which means higher sensitivity between the reflectivity at these five bands and soil salt content. Thus, the reflectivity at these five bands is the best choice to inverse the water-soluble salt content of saline soil in the protection zone.

#### 3.2.2. Multiple Linear Regression Model

As a traditional scientific method with a strong application, regression analysis can be used to confirm the interdependently quantitative relationship among two or more variables. Now, it has been widely applied in various scientific fields. In practice, one phenomenon is always related to multiple elements, so there emerges the most optimal combination of multiple independent variables for the prediction or measurement of dependent variables. By contrast, this is more effective and more practical than the method, which only takes advantage of one independent variable [23]. Therefore, this research adopted a multiple linear regression model to simulate the correlation between the soluble salt content of soils at 10 training sample points and the reflectivity of synchronous remote sensing images at sensitive bands on basis of SPSS software. Finally, the obtained regression formula is shown in the following:

y = 23.25 × B_2_ − 119.43 × B_6_ + 375.46 × B_7_ − 123.36 × B_8_ − 237.96 × B_8A_ + 31.20 (6)

where, y means the soluble salt content of soils, and B_2_, B_6_, B_7_, B_8_ and B_8A,_ respectively represent the reflectivity at 2, 6, 7, 8 and 8A bands.

#### 3.2.3. BP Neural Network Inversion Model

The soil spectrum is the result of many factors, and the inversion of the soluble salt content of soils is complex. As a technology developed in recent years, the BP neural network has a strong nonlinear mapping ability. Therefore, in this research, a BP neural network model was used to inverse the soluble salt content of soils in the reserve, and the results were compared with the results of the multiple linear regression model.

Such as the multiple linear regression model, the reflectance of Band 2, Band 3 and Band 4 of 10 bands pretreated Sentinel-2A-MSI images is used as the input of the BP neural network model. Additionally, the soluble salt content of soils of 10 training samples is used as the output of the neural network. Before the network training, the input and output data of the neural network are normalized and standardized, and the expected error is set to 0.001.

Considering the practical problems of this research, a single hidden layer model is used in the design of the BP neural network. In this research, the number of hidden layer nodes is determined by the step-by-step method. That is, the number of hidden layer elements is set to two first, and if it does not meet the requirements, the number of hidden layer nodes is gradually increased until it is appropriate. Through repeated experiments, the final number of nodes in the hidden layer was determined to be 14. The simulation results of the neural network model are shown in Table 5.

#### 3.2.4. Salinity Result and Analysis

In this study, the model accuracy is analyzed with the soluble salt content of soil data of the other 10 samples. In Table 5, among prediction results provided by the multiple linear regression model, relative error ranges from 44.34% to 98.88%. In this way, it is convenient to the inverse soluble salt content of soils through the established multiple linear regression model on the basis of Sentinel-2A remote sensing images, but the precision of the inversion result is still at a low level. However, the maximum relative error of the soluble salt content of soils inversed by the BP neural network model is 80.90%, and the minimum relative error is −4.00%. Compared with the multiple linear regression model, the inversion accuracy of the BP neural network model has been significantly improved.

**Figure 4:**
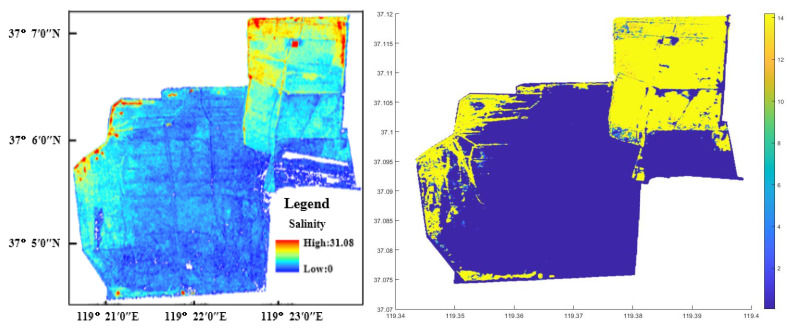
The distribution of soluble salt content of soils by multiple linear regression model in study area based on multiple linear regression model (**left**) and BP neural network inversion model (**right**).

Figure 4 demonstrates the distribution consequence of the soluble salt content of soils through the inversion in the study area based on a multiple linear regression model and the BP neural network inversion. From the figure, the soluble salt content of soils increases along the direction of land to sea, which is in line with the real situation. This shows that it is feasible based on an appropriate inversion model and Sentinel-2A multispectral images to inverse the soluble salt content of soils.

### 3.3. Wetness

#### 3.3.1. Tasseled Cap Transformation

The tasseled cap transformation, also named the Kauth–Thomas transformation, is a linear transformation method where coordinate space rotation happens. Through multidimensional rotation, the method can generate new components, but only the first three of them have clear physical significance [24,25]. The first component is brightness, which reflects the comprehensive effect of the overall reflectance and is only related to the physical process that affects the overall reflectance, mainly reflecting the radiation level in the infrared band. The second is greenness. It is a kind of comprehensive response to the absorption of plant photosynthesis in the visible light band and the strong plant reflection in the near-infrared band, so it has a close relationship with vegetation coverage, leaf area index and biomass. For the third, it is wetness. It is the difference between the total reflection energy of the visible light band and the near-infrared band and the reflected energy in the short-wave infrared band. Thus, the third component displays ground moisture conditions, especially the moisture state of the soil. 

Currently, the components of brightness, greenness and wetness obtained through tasseled cap transformation have been widely utilized in eco-environment monitoring. For wetness, it can reflect the moisture of vegetation and soil, so it is closely related to eco-environment conditions. Therefore, the wetness index in this research was represented by the third component provided through the tasseled cap transformation of the remote sensing image [26]. 

The following shows the tasseled cap transformation formulas of the Sentinel-2A satellite and Landsat satellite for wetness component calculation.

Wetness(Sentinel-2A) = 0.1509 × B_2_ + 0.1973 × B_3_ + 0.3279 × B_4_ + 0.3406 × B_8_ − 0.7112 × B_11_ − 0.4572 × B_12_ (7)

Wetness(Landsat) = 0.1509 × B_Blue_ + 0.1973 × B_Green_ + 0.3279 × B_Red_ + 0.3406×B_NearInfrared_ − 0.7112 × B_SWIR-1_ − 0.4572 × B_SWIR-2_ (8)

In Formulas (7) and (8), the coefficients correspond to the reflectivity values at 2, 3, 4, 8, 11 and 12 bands of Sentinel-2A satellite and the atmospheric apparent reflectivity of the Landsat satellite, respectively. 

#### 3.3.2. Wetness Result Analysis

**Figure 5:**
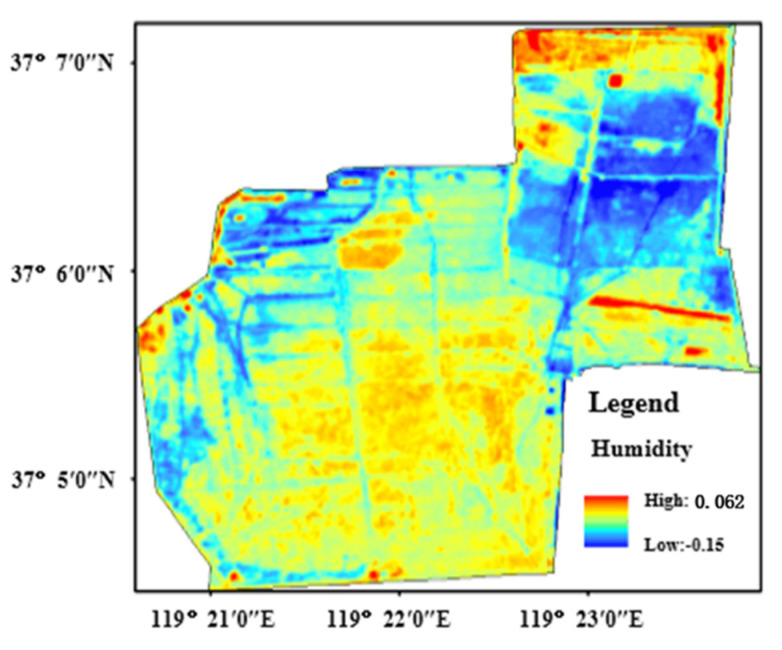
The distribution of wetness in study area.

Figure 5 demonstrates the wetness distribution characteristic of the protection zone using the remote sensing data of the Sentinel-2A satellite. Wetness in the protection zone is within the range from −0.15 to 0.062. The region with high vegetation coverage has higher wetness in the *Tamarix chinensis* forest besides special coverage areas of the marginal areas of coastal beach and internal pool. The region with low wetness is the low-coverage *Tamarix chinensis* area. On the whole, the spatial distribution characteristics of wetness were consistent with the spatial distribution characteristics of vegetation coverage.

### 3.4. Dryness

**Figure 6:**
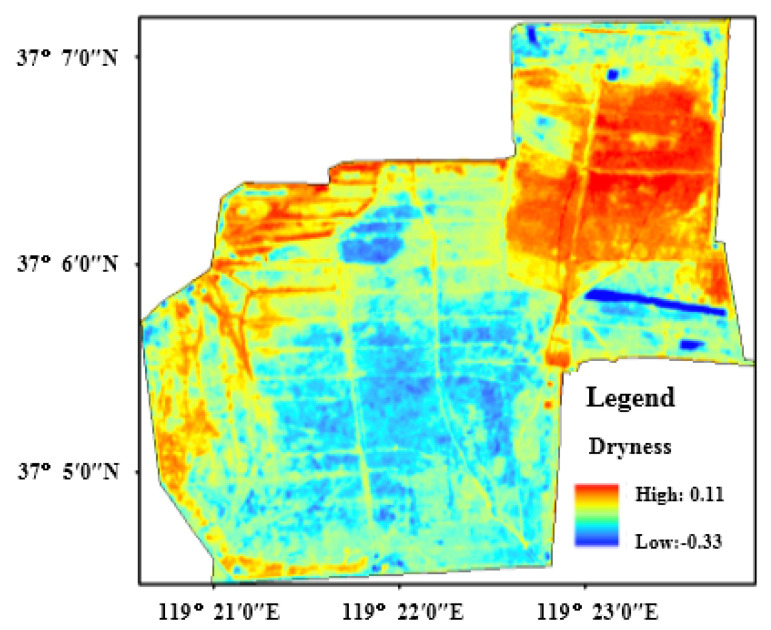
The distribution of dryness in study area.

Surface drought is one of the main factors causing eco-environment deterioration in the protection zone [27]. Therefore, in this study, the dryness of RSEI is represented by the bare soil index (SI), which can characterize the bare surface condition [28]. The SI formula is shown in the following:

(9) $$\mathrm{SI}=\frac{\left(\rho 11+\rho 4\right)\left(\rho 8+\rho 2\right)}{\left(\rho 11+\rho 4\right)+\left(\rho 8+\rho 2\right)}$$

where, ρ2, ρ3, ρ4, ρ8 and ρ11, respectively, point to the reflectivity of the Sentinel-2A remote sensing image at 2, 3, 4, 8 and 11 bands. Figure 6 shows the dryness distribution characteristic of the protection zone. Dryness is exactly inverse to wetness in the spatial distribution characteristic. That is to say, high dryness is found in the north, the northeast and the west of the *Tamarix chinensis* forest, which is low in vegetation coverage. Furthermore, low dryness appears in the coastal area of *Tamarix chinensis* forest, internal pools, the surrounding southern water system and the highly covered *Tamarix chinensis* area.

### 3.5. Greenness

**Figure 7:**
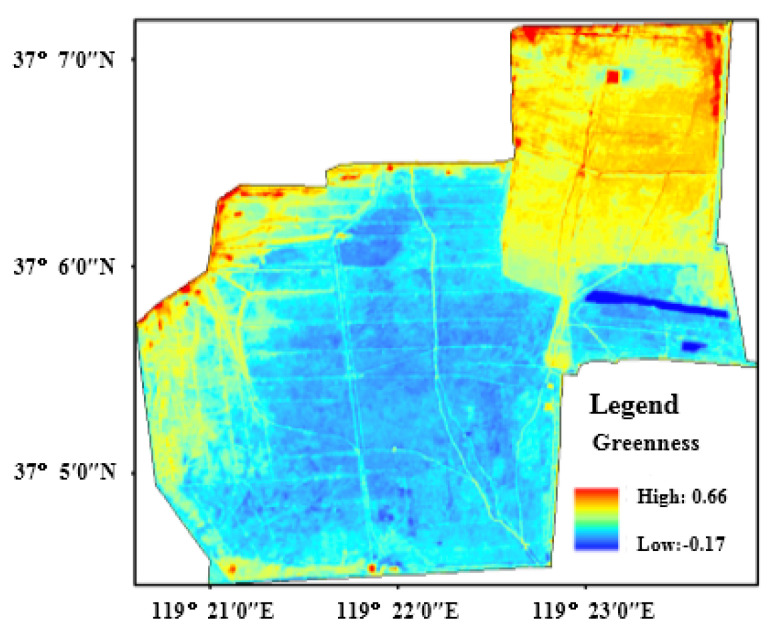
The distribution of greenness in study area.

The normalized difference vegetation index (NDVI) is a well-known broad-band greenness index, which is often used in vegetation productivity modeling. In addition, as the most widely used vegetation index, NDVI is closely related to plant biomass, leaf area index and vegetation coverage [29]. Therefore, it was selected to represent the greenness of the RSEI evaluation system in this research. In Figure 7, which contains the greenness distribution in the protection zone, NDVI is high in most regions, lower in roads and the marginal region, and the lowest in the northeast area and the near beach area.

### 3.6. Heat

This research chose surface temperature as the heat index in the RSEI evaluation system. Until now, the surface temperature inversion algorithms, depending on the thermal infrared remote sensing, have included a single algorithm (including an atmospheric correction algorithm based on the radiation transfer equation), a multichannel algorithm, a multiangle algorithm, a multitemporal algorithm and a hyperspectral inversion algorithm [30]. To realize the surface temperature inversion in the protection zone, this research took advantage of the atmospheric correlation algorithm based on the real detection data of the atmospheric profile. Since the Sentinel-2A remote sensing image does not have a thermal infrared band. This research selected the thermal infrared band of the Landsat8-TIRS image, similar to the Sentinel-2A image in imaging time, to calculate the heat index of the protection zone. In order to match the 10 m resolution of the Sentinel-2A image and improve the evaluation results, the temperature results with the Landsat8-TIRS image at 30 m resolution were resampled to 10 m resolution. It should be noted that, during radiation calibration of the Landsat8-TIRS image, Band 11 was sensitive to error, which caused a larger deviation. Thus, this research chose Band 10 to extract the surface temperature of the protection zone [31].

**Figure 8:**
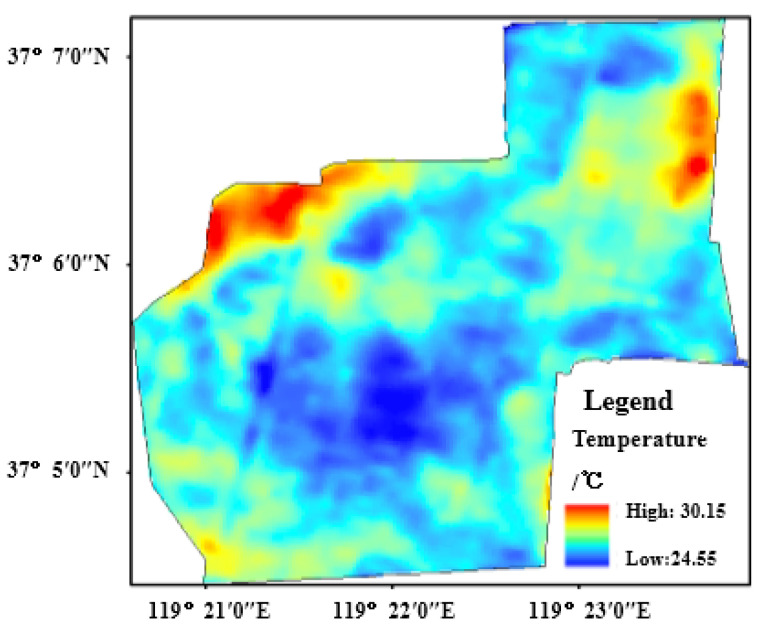
The distribution of temperature surface in study area.

In Figure 8, the temperature in the protection zone ranges from 24.55 °C to 30.15 °C at 10:00 a.m. on 26 September 2019. Moreover, the surface temperature is low in the middle region with highly covered *Tamarix chinensis* and is high in the south and the north. Especially in the north, the surface temperature is relatively higher because most of the area is the region of resource recovery, and its *Tamarix chinensis* is low in density.

## 4. Comprehensive Evaluation of Eco-Environment in Protection Zone

### 4.1. Ecological Factor Normalization

Through the above methods or models, it is possible to obtain the result of a single evaluation index used for the eco-environment evaluation in the protection zone. However, because factors have different dimensions, it is hard to directly couple factors to comprehensively evaluate the eco-environment. To avoid the weight imbalance caused by different dimensional units, it is essential to normalize all factors and then calculate and analyze the comprehensive ecological index of the protection zone [32]. In the consideration of unavoidable noise in remote sensing images, the statistical maximum and minimum are not necessarily effective. That is the reason why this research first set 2% confidence intervals in the pixel histogram of the statistical evaluation index, and then all ecological factors were normalized by the normalization formula.

### 4.2. The Analysis Evaluation Method of Eco-Environment Quality-SPCA

Among many evaluation methods for natural eco-environments, spatial principal component analysis (SPCA) transforms related multivariable spatial data into a few comprehensive factors, among which the correlation is low. The SPCA is based on the rotation of spatial coordinate axes of the characteristic spectrum and the orthogonal linear variation of multiple variables. This method can maximize the retention of the initial information with fewer comprehensive factors [33]. Therefore, it is not necessary for SPCA to make sure of the weight of every index; therefore, deviation of the result can be prevented from the set weight by different expers and methods. The impact of each index on RSEI is completely determined by the raw data of index parameters. Therefore, the obtained RSEI ecological index can reflect the ecological environment of the whole region.

In this study, the standardized data in the evaluation index system, including AOD, greenness, wetness, dryness, heat, and salinity, was processed by SPCA. The RSEI of the study area was calculated, and the spatial characteristics and the spatiotemporal evolvement of the eco-environment were analyzed.

### 4.3. The Eco-Environment Quality Analysis of Protection Zone Based on RSEI (September 2019)

Table 6 includes the statistical and calculation results of the mean value, the standard deviation of all factors and RESI in September 2019. From the table, the mean RESI is 0.61, which proves that the overall eco-environment quality is at a good level (Please see the following Section 4.4 for the reason why this value is a good level). In terms of the contribution of all factors to PC1 (RESI) in Table 7, greenness and wetness, which are positively related to the eco-environment, are positive values. However, dryness, temperature, AOD and salinity, which are negatively related to the eco-environment, are negative values. This is consistent with the positive effect of greenness and wetness and the negative effect of dryness and heat on the eco-environment in practice. At the same time, it also illustrates that vegetation restoration is able to increase the greenness and wetness of eco-environment quality in protection zones [34].

Through further analysis in Table 7, it is found that the contribution of the characteristic value of PC1, PC2 and PC3 accounts for 56.95%, 24.7% and 7.95%, respectively, among the RESI statistical results on the basis of the PCA model. The cumulative contribution rate of these three characteristic values reached 89.6%, more than 85%. This explains that the correspondingly representative information of principal components is able to represent most eco-environment quality information, and it is reasonable to evaluate eco-environment with RSEI through SPCA. Thus, it is possible to use RSEI and the derived statistical information for the follow-up eco-environmental evaluation and analysis in the protection zone [35].

This research analyzed the correlations among six factors and the correlations between these factors and RSEI. As reported in Table 8, the correlation between greenness and the other factors appears to be the strongest correlation, with a mean coefficient of 0.51. Additionally, the mean correlation between RSEI and the other factors is 0.62, 19.60% higher than the strongest correlation of the former. Thus, it can be seen that RSEI integrates each component’s information well and can more comprehensively reflect the eco-environment condition of the protection area [36].

**Figure 9:**
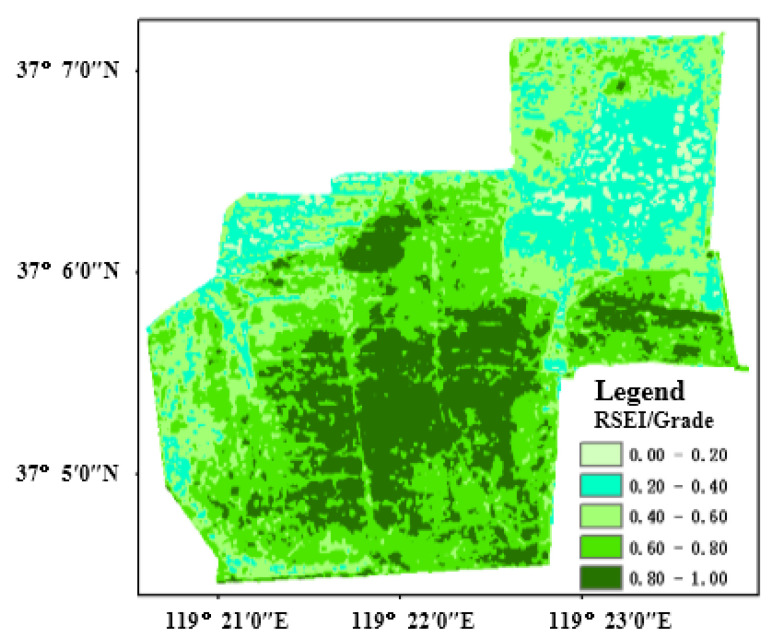
Spatial distribution and classification of RSEI in study area (9 September 2019).

The spatial distribution of the eco-environment quality of the protection zone in September 2019 is shown in Figure 9, where the south is better than the north, and the middle is superior to the surroundings. In detail, the obviously excellent eco-environment quality appears in the middle protection zone, and the worst is mainly located in the western environmental management area and the northern resource recovery area. To clearly demonstrate the spatial distribution of eco-environment quality, the research divided the values of RSEI in September 2019 into five grades at an internal of 0.2. According to RSEI values from large to small, the five grades corresponded to excellent, good, medium, worse and worst. After statistics, their proportions were respectively 22.62%, 32.23%, 22.95%, 16.68% and 2.62%, and the excellent and good rate was 54.89%. In this way, the result proved that the overall eco-environment quality of the protection zone is generally at a good level.

### 4.4. The Evolvement Analysis of Spatiotemporal Pattern of RSEI (2000–2019)

To quantitatively reflect the spatiotemporal pattern evolvement of eco-environment in protection zone from 2000 to 2019, this research selected four time nodes, when representative events (see Table 1) happened, respectively in 2000, 2007, 2014 and 2019. Because there was a limitation to acquiring and verifying the historical remote sensing data of AOD and salinity, these two factors were not contained in the analysis of spatiotemporal pattern evolvement. This research also obtained PC1 and the mean RSEI of the eco-environment quality index in the protection zone at the above four time nodes through the same method. In Table 9, the mean RSEI of the protection zone shows an increasing trend, from 0.33 in 2000 to 0.58 in 2019. It is worth noting that RSEI grew by 0.16 during 2007–2014 and 0.10 during 2014–2019.

To more intuitively show the advantages and disadvantages of the eco-environment in the protection zone, the RSEI values at those four time nodes were divided into five grades at an internal of 0.2. According to values from large to small, the five grades corresponded to excellent, good, medium, worse and worst. From the area statistics of five grades in Table 10, it could be found that the excellent and good rate was 4.05% in 2000, 4.55% in 2007, 49.42% in 2014 and 55.33% in 2019. Besides, the excellent and good rates increased by 44.87% from 2007 to 2014, meaning the significant effect of ecological restoration during this period.

Table 11 shows the dynamic change of RSEI corresponding to three different time periods of the protection zone. During 2000–2007, RSEI showed a reducing trend. From the perspective of dynamic changes in the eco-environment, the deteriorative area reached 7.42 km^2^, 47.93% of the protection zone area, but the improved area was only 3.22 km^2^. From 2007–2014, the area with decreasing RSEI was only 2.90 km^2^, 18.75% of the protection zone area, and by contrast, the area with improved ecological conditions was 11.29 km^2^, 72.94% of the protection zone area. From 2014–2019, the area with decreased RSEI reached 3.21 km^2^ and the area with increased RSEI was 9.17 km^2^, which respectively occupied 18.75% and 72.94% of the total area.

**Figure 10:**
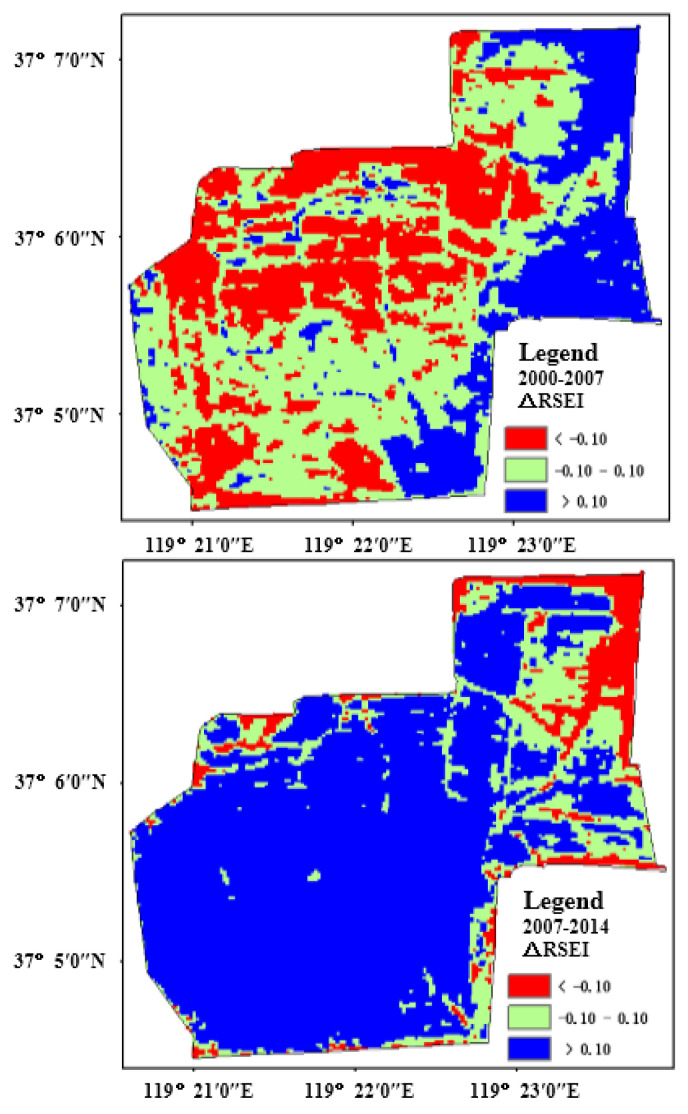

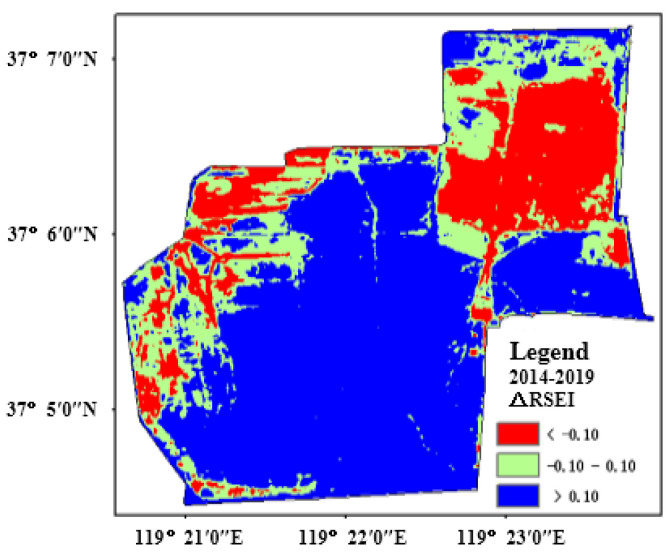
The spatiotemporal changes of eco-environment in study area.

To intuitively and comprehensively demonstrate the spatiotemporal change of eco-environment in the protection zone, the change detection was conducted towards the RSEI images at those four time points by the image difference method. In Figure 10, red represents the deteriorative ecological area (ΔRESE < −0.1), blue represents the improved ecological region (ΔRESE > 0.1), and green represents the stable ecological region (−0.1 ≤ ΔRESE ≤ 0.1). By analyzing Figure 10, during 2000–2007, the improved region is mainly distributed in the east of the protection zone, and the deteriorative are scattered in the protection zone on a large scale.

During the period of 2007–2014, the eco-environment gradually improved as a general trend, and the deteriorative areas are mainly distributed in the northwest and dispersedly distributed in the marginal region. During 2014–2019, eco-environment still keeps an improving trend, especially, in the middle region, and the deteriorative region is in the northwest in the comparison with the situation from 2007 to 2014. In general, the eco-environment quality has been greatly improved through ecological restoration over the last 14 years.

## 5. Conclusions

To monitor and evaluate the eco-environment effect of ecological restoration on the *Tamarix chinensis* forest in the protection zone, this research built a set of comprehensive evaluation systems for eco-environment quality by remote sensing technology. On the method or model, DDV, multiple linear regression model, BP neural network inversion model, tasseled cap transformation and atmospheric correction algorithm were applied in this study. Finally, the conclusions are as follows:

(1) The RSEI system included AOD, salinity, greenness, wetness, heat and dryness, respectively. Since the study area belongs to the coastal *Tamarix chinensis* wetland, and its land cover is of forest type. The factors of salinity and AOD were selected as the evaluation index systems. The last four factors are not only important factors of natural ecological quality but also are closely related to human activities. Therefore, the RSEI system was scientific and reasonable;

(2) The correspondingly representative information of principal components is able to represent most eco-environment quality information, and it is reasonable to evaluate eco-environment with RSEI through SPCA;

(3) The spatial distribution of eco-environment quality of the protection zone in September 2019 is that the south is better than the north, and the middle is superior to the surroundings;

(4) The eco-environment quality of the protection zone shows an increasing trend from 2000 to 2019, especially from 2007 to 2014 and 0.10 during 2014–2019. This shows that the ecological restoration of the protection zone is meaningful.

## Figures and Tables

**Table 1:** Time, category and basis of remote sensing image selection.

Time	Image Category	Basis of Image Selection
9/2000	Landsat-4/5-TM	Original state of the reserve
9/2007	Landsat-5-TM	In 2007, the State Oceanic Administration approved the establishment of the reserve
9/2014	Landsat-8-OLI/TIRS	The begins of ecological restoration in reserve with large scale
9/2019	Landsat-8-OLI/TIRS	More than 200 hm^2^ Tamarix forest repaired
9/2019	HJ1-CCD	AOD inversion
9/2019	Sentinel/2A/MSIL1C	RSEI of current reserve

**Table 2:** The lookup table of AOD for HJ-1-CCD data(part).

$\rho_{\alpha }$	T	*S*	$\mathrm{Solar}\;\mathrm{Zenith}/\theta_{s}$	$\mathrm{Satellite}\;\mathrm{Zenith}/\theta_{v}$	Relative Azimuth/Ø	AOD-τ
0.13766	0.83489	0.06554	0	0	0	0.00010
0.13766	0.83489	0.06554	0	0	12	0.00010
0.13766	0.83489	0.06554	0	0	24	0.00010
0.13766	0.83489	0.06554	0	0	36	0.00010
0.17874	0.73011	0.08739	6	3	48	0.25
0.17874	0.73011	0.08706	6	3	60	0.25
0.17874	0.73011	0.08671	6	3	72	0.25
0.17874	0.73011	0.08637	6	3	84	0.25
0.26870	0.28913	0.17870	24	30	96	1.50
0.26870	0.28913	0.17731	24	30	108	1.50
0.26870	0.28913	0.17640	24	30	120	1.50
0.26870	0.28913	0.17588	24	30	132	1.50
0.28252	0.12120	0.32403	60	60	144	1.95
0.28252	0.12120	0.33546	60	60	156	1.95
0.28252	0.12120	0.34339	60	60	168	1.95
0.28252	0.12120	0.34623	60	60	180	1.95

**Table 3:** Correlation coefficient between reflectivity (Sentinel-2A) and salinity.

Sentinel-2A Bands	Band 2-Blue	Band 3-Green	Band 4-Red	Band 5-Vegetation Red Edge	Band 6-Vegetation Red Edge
Central wavelength/μm	0.49	0.56	0.665	0.705	0.74
Correlation coefficient/Ri	0.22	0.09	0.38	0.03	−0.59
Mean square deviation/σi	107.28	144.97	268.45	224.85	302.28
Sentinel-2A Bands	Band 7-Vegetation Red Edge Band	Band 8-NIR Band	Band 8A-Vegetation Red Edge Band	Band 11-SWIR Band	Band 12-SWIR Band
Central wavelength/μm	0.783	0.842	0.865	1.61	2.19
Correlation coefficient/Ri	−0.59	−0.59	−0.61	0.31	0.46
Mean square deviation/σi	330.19	312.58	365.47	402.60	463.91

Note: Band 1, Band 9 and Band 10 are aerosol monitoring band, water vapor monitoring band an ocean current monitoring band, respectively. Because these three bands are low in resolution, so there does not exist correlation calculation between reflectivity of these bands and the soluble salt content of soils [21].

**Table 4:** Diagnostic index of reflectivity with each band (Sentinel-2A).

Band	Band 2	Band 3	Band 4	Band 5	Band 6	Band 7	Band 8	Band 8A	Band 11	Band 12
|Di|	0.20	0.06	0.14	0.01	0.20	0.18	0.19	|−0.17|	0.08	0.10

**Table 5:** Verification of the inversion results of soluble salt content of soils by multiple linear regression model and BP neural network model.

No.	Soluble Salt Content of Soils-Measured g/Kg	Multiple Linear Regression Model			BP Neural Network Inversion Model		
		Soluble Salt Content of Soils-Inversion g/Kg	Error g/Kg	Relative Error %	Soluble Salt Content of Soils-Inversion g/Kg	Error g/Kg	Relative Error %
1	8.18	14.70	6.52	44.34	8.771	0.735	8.99
2	1.53	10.96	9.43	86.04	1.490	0.068	4.47
3	12.4	6.89	−5.51	−79.95	1.660	1.592	12.84
4	0.08	3.12	3.04	97.43	0.077	−0.003	−4.00
5	0.22	4.60	4.38	95.22	0.129	−0.091	−41.59
6	0.14	3.26	3.12	95.71	0.151	0.011	7.78
7	0.07	4.37	4.30	98.40	0.134	0.057	80.90
8	0.04	3.56	3.52	98.88	0.008	0.014	34.27
9	0.06	2.98	2.92	97.99	0.027	−0.017	−28.92
10	0.08	3.61	3.53	97.78	0.042	−0.038	−47.44

**Table 6:** The statistics of mean, standard deviation and RESI for study area.

Index	Mean	Standard Deviation
Greenness	0.32	0.11
Wetness	−0.046	0.029
Dryness	−0.064	0.069
Temperature	26.60	0.82
Salinity	6.72	4.26
AOD	0.14	0.23
RSEI	0.61	0.21

**Table 7:** The analysis result by SPCA in study area.

Index	PCA					
	PC1	PC2	PC3	PC4	PC5	PC6
Greenness	0.31	0.27	0.53	0.33	0.26	0.61
Wetness	0.65	0.35	−0.34	0.37	0.10	−0.44
Dryness	−0.31	0.44	0.34	−0.25	0.57	−0.45
Temperature	−0.06	−0.48	−0.38	0.02	0.77	0.17
Salinity	−0.45	0.20	−0.12	−0.83	0.00	0.24
AOD	−0.42	−0.58	0.58	−0.11	−0.02	−0.38
Eigenvalue	0.30	0.13	0.04	0.04	0.01	0.00
Contribution rate of eigenvalue/%	56.95	24.70	7.95	7.27	2.42	0.70

**Table 8:** Correlation matrix of each factor and RSEI.

Index	Greenness	Wetness	Dryness	Salinity	AOD	Temperature	RSEI
Greenness	1.00	0.64	−0.39	−0.26	−0.41	−0.34	0.28
Wetness	0.64	1.00	−0.01	−0.01	−0.01	−0.01	0.39
Dryness	−0.39	−0.01	1.00	0.00	0.01	0.01	−0.93
Salinity	−0.26	−0.01	0.00	1.00	0.01	0.00	−0.47
AOD	−0.41	−0.01	0.01	0.01	1.00	0.01	−0.34
Temperature	−0.34	−0.01	0.01	0.00	0.01	1.00	−0.96
Mean	0.51	0.28	0.24	0.21	0.24	0.23	0.62

Note: the mean value of correlation coefficient is calculated by the absolute value of the correlation between one index and other indexes.

**Table 9:** PC1 and RSEI (Mean) of 4 nodes in study area.

Year	PC1				RSEI/Mean
	Greenness	Wetness	Dryness	Temperature	
2000	0.44	0.73	−0.16	0.50	0.33
2007	0.65	0.24	−0.43	0.58	0.32
2014	0.60	0.32	0.59	−0.43	0.48
2019	0.55	0.77	0.09	0.31	0.58

**Table 10:** The area/km^2^ and ratio/% of ecological grade in study area.

RSEI Grade	2000		2007		2014		2019	
	Area	Ratio	Area	Ratio	Area	Ratio	Area	Ratio
Worst (0–0.2)	2.15	13.91	3.77	24.34	0.04	0.27	1.26	8.17
Worse (0.2–0.4)	8.99	58.07	7.06	45.61	1.37	8.86	2.40	15.49
Medium (0.4–0.6)	3.71	23.98	3.95	25.50	6.42	41.44	3.25	21.01
Good (0.6–0.8)	0.47	3.03	0.66	4.27	5.83	37.66	5.57	35.96
Excellent (0.8–1.0)	0.16	1.02	0.04	0.28	1.82	11.76	3.00	19.37
Total	15.48	100%	15.48	100%	15.48	100%	15.48	100%

**Table 11:** The area/km^2^ and ratio/% of RSEI of dynamic change.

Time Range	2000–2007		2007–2014		2014–2019	
Type	Area	Ratio	Area	Ratio	Area	Ratio
Worse (RSEI < −0.10)	7.42	47.93	2.90	18.75	3.21	20.71
Unchanged (0.10 ≥ RSEI ≥ 0.10)	4.84	31.26	1.29	8.31	3.10	20.02
Better (RSEI > 0.10)	3.22	20.81	11.29	72.94	9.17	59.27
Total	15.48	100.00	15.48	100.00	15.48	100.00

## Data Availability

The study did not report any data.
